# Phantom for standardization in functional near-infrared spectroscopy, part 1: implementation and usage

**DOI:** 10.1117/1.NPh.12.4.045010

**Published:** 2025-12-05

**Authors:** Hiroshi Kawaguchi, Yukari Tanikawa, Toru Yamada, Marianne Floor-Westerdijk, Miriam van der Hoek, Lin Yang, Alexander von Lühmann, Patrick Britz, David R. Busch, Alessandro Torricelli, Antonio Pifferi, Dirk Grosenick, Heidrun Wabnitz

**Affiliations:** a National Institute of Advanced Industrial Science and Technology, Tsukuba, Japan; b Artinis Medical Systems, Elst, The Netherlands; c NIRx Medizintechnik, Berlin, Germany; d Physikalisch-Technische Bundesanstalt, Berlin, Germany; e Technische Universität Berlin, Berlin, Germany; f Berlin Institute for the Foundations of Learning and Data, Berlin, Germany; g University of Texas Southwestern Medical Center, Department of Anesthesiology & Pain Management, Biomedical Engineering, Neurology, Dallas, Texas, United States; h Politecnico di Milano, Dipartimento di Fisica, Milan, Italy; i Istituto di Fotonica e Nanotecnologie, Consiglio Nazionale delle Ricerche, Milan, Italy

**Keywords:** functional near infrared spectroscopy, international standard, phantom, diffuse attenuation, performance tests

## Abstract

**Significance:**

The use of functional near-infrared spectroscopy (fNIRS) is expanding, including examples of devices approved for routine clinical use for specific indications. Instrument standardization is a critical element to facilitate clinical adoption. Currently, there is no published description of a phantom that meets the requirements of IEC 80601-2-71, the IEC/ISO standard for fNIRS equipment.

**Aim:**

We report an example of the implementation of the fNIRS standard phantom described in IEC 80601-2-71. We further report results from measurement campaigns with this phantom prototype in Japan and Europe.

**Approach:**

A phantom prototype was built and circulated among multiple academic and commercial fNIRS groups with diverse instrumentation. The phantom prototype was characterized by wavelength-dependent measurements of its diffuse optical features and utilized in a test for fNIRS signal magnitude as described in IEC 80601-2-71.

**Results:**

The optical loss of this phantom prototype as well as its change by varying an internal aperture were found to be ∼40  dB and between 3.5 and 3.6 dB, respectively. The test for fNIRS signal magnitude based on this phantom was successfully carried out on various commercial fNIRS devices.

**Conclusions:**

The characteristics of the phantom prototype agreed with the specifications in the standard. The phantom prototype proved suitable for the related test in IEC 80601-2-71.

## 1 Introduction

Functional near-infrared spectroscopy (fNIRS, functional NIRS) records localized changes in the concentrations of oxy- and deoxyhemoglobin that are related to neuronal activation of the human brain. As near-infrared (NIR) light (650 to 950 nm) is minimally absorbed by tissue, this technique may be noninvasive: a small spot on the scalp is illuminated and diffuse light is detected after passage through tissue. With a typical source-detector separation of a few centimeters (e.g., 3 cm), the detected light has penetrated deeply enough into the tissue to carry information about haemodynamic processes in the cerebral cortex. The spectral differences between oxy- and deoxyhemoglobin in the NIR region permit calculation of changes in their concentrations from changes in detected light intensity at two or more wavelengths. fNIRS devices can be compact and portable, enabling measurements in a wide range of locations and subject postures, and thus studies of brain activation in naturalistic environments. These capabilities have in turn led to a variety of fNIRS applications in human neuroscience and medicine.^1^^–^^6^

Clinical trials and studies have been reported in healthy subjects, for multiple disease states, and for a variety of physiological conditions. The utility of fNIRS is perhaps greatest in subjects and situations where other neuroimaging techniques are difficult to apply, e.g., pediatrics,^7^^–^^10^ motor rehabilitation,^11^^–^^13^ and gait studies.^14^ In addition, fNIRS-based quantitative biomarkers may complement or replace existing subjective assessment methods, e.g., in psychiatry,^15^^–^^18^ anesthesiology,^19^^,^^20^ and neurology,^21^^,^^22^ for diagnosis, symptom grading, therapeutic efficacy assessment, and prognosis prediction. Several fNIRS devices have been approved or certified by the Pharmaceuticals and Medical Devices Agency (PMDA) of Japan. Reimbursement by the Japanese public medical insurance system is provided for their use in preoperative testing for neurosurgery and in assisting in the differential diagnosis of depressive states.^23^

Because standardization is a critical element to enable clinical adoption, Japanese manufacturers collaborated to establish a domestic standard for fNIRS devices. Subsequently, the development of this international standard was proposed by Japan in 2011. The international standard “Particular requirements for the basic safety and essential performance of functional near-infrared spectroscopy (NIRS) equipment,” IEC 80601-2-71^24^ has been jointly developed by the International Electrotechnical Commission (IEC) and the International Organization for Standardization (ISO). It is a particular standard in the 60,601 family of standards for medical electrical equipment. It covers continuous-wave (CW) fNIRS devices, whereas time- and frequency-domain fNIRS devices as well as tissue oximeters are excluded from its scope. The first edition of IEC 80601-2-71 was published in 2015 as a result of the consensus-based process of development of international standards pursued by the IEC/ISO Joint Working Group (JWG) “Oximeters” (IEC/TC 62/SC 62D/JWG 5 and ISO/TC 121/SC 03/JWG 10). In 2025, following a systematic review, the second edition of this standard was published.^24^ For the history of the development of the standard, refer to the literature.^25^

The fNIRS standard IEC 80601-2-71 includes several quantitative performance tests aimed at ensuring safe and reliable performance of medical fNIRS equipment. Most of these tests rely on a specific turbid phantom, here termed “fNIRS standard phantom.” It provides a realistic overall diffuse attenuation in relation to fNIRS measurements on the human head and is equipped with a mechanism to change the attenuation by a specific amount. The prominent purpose of this phantom is to implement the main test in the standard, i.e., the test for the fNIRS signal magnitude (“pathlength-dependent hemoglobin change”). Other phantom-based tests included in the fNIRS standard address signal-to-noise ratio, signal stability, response time, and signal cross-talk. Section 6.1 in Appendix cites several definitions of terms and specifications of the phantom from IEC 80601-2-71:2025^24^ that are relevant for the present publication.

The prototype of the fNIRS standard phantom described herein was developed in a collaboration between the National Institute of Advanced Industrial Science and Technology (AIST) and several Japanese fNIRS manufacturers, in the process of preparing the first edition of the fNIRS standard (2010–2011). In this publication, it will be termed “fNIRS standard phantom prototype,” in short “phantom prototype.” Note that the basic optical concept of this kind of phantom was previously presented in the work by Firbank and Delpy at University College London in 1994, with the purpose of checking the long-term stability and accuracy of relative attenuation measurements in fNIRS.^26^

Two measurement campaigns have been conducted with the fNIRS standard phantom prototype. In the first campaign, replicas of this prototype were shared with several Japanese fNIRS manufacturers (Hamamatsu Photonics K. K.; Hitachi, Ltd; Shimadzu Corp.). As a trial, performance tests were run on their fNIRS equipment to check the practical aspects of the suitability of the phantom prototype.

A second phantom measurement campaign was conducted in 2022/2023 during the revision of the fNIRS standard, among members of the JWG “Oximeters.” This campaign was held in Europe, under the leadership of AIST, as a round-robin campaign circulating one of the original (i.e., 2010) fNIRS standard phantom prototypes to a total of six partners. These included two manufacturers of CW fNIRS devices (Artinis Medical Systems NV, The Netherlands; NIRx Medizintechnik, GmbH, Germany), two research institutions (PTB Berlin, Germany, and Politecnico di Milano, Italy), a time-domain NIRS device manufacturer (PIONIRS s.r.l., Italy), and a phantom manufacturer (BioPixS Ltd., Ireland). The purpose of this second campaign was threefold:

(i) During the revision of the standard, it was important for the European manufacturers to gain experience in testing their devices with the phantoms that had so far not been available outside Japan.
(ii) A basic characterization of the phantom was performed with respect to optical parameters that are specified in the standard, i.e., the total optical loss, the change in optical loss, and their wavelength dependence.
(ii) For a deeper understanding of the phantom’s function, the optical characteristics of the phantom were investigated in more detail. The results of the estimation of the wavelength-dependent optical properties (scattering and absorption) of the phantom material and of dedicated Monte-Carlo simulations of light propagation to model the characteristics of diffuse attenuation of the composite phantom prototype are presented in a companion publication.^27^ Items (i) and (ii) are covered in the present publication.

The purpose of the present paper is to disseminate a practical implementation of the fNIRS standard phantom based on the specifications defined in the standard IEC 80601-2-71, as well as the estimation of its optical attenuation characteristics that are relevant for the performance tests of fNIRS equipment included in this standard. To date, there has been no publication detailing the concrete implementation of the phantom specified in the international standard, and we consider this information to be crucial for readers in the fNIRS community.

## 2 Materials and Methods

### 2.1 Concept and Design of the fNIRS Standard Phantom

The standard IEC 80601-2-71 includes various tests to evaluate the performance of fNIRS equipment and employs a phantom with known and stable optical characteristics to implement these tests in an accurate and reproducible manner. The standard defines and specifies a “functional NIRS phantom” (see Sec. 6.1 in Appendix) that is required for several performance tests.

The design of the fNIRS standard phantom must meet two goals: (i) to mimic the diffuse character of light emerging from the human head, with a typical total attenuation, and (ii) to mimic attenuation changes due to changes in oxy- and deoxyhemoglobin concentrations caused by brain activation. Section 6.2 in Appendix lists the specifications of the fNIRS phantom as provided by the fNIRS standard. According to these specifications, the fundamental components of the phantom are two turbid cylindrical attenuators and two switchable circular apertures of different diameters sandwiched between the attenuators. The specifications of the turbid attenuators include limits on their dimensions, absorption, reduced scattering coefficients, and surface roughness. Moreover, the material is required to be optically homogeneous and isotropic. For the purpose of the tests in the fNIRS standard, it is not relevant to mimic details of light propagation in the human head with its complex tissue structure and optical properties.

Figure 6 in Sec. 6.2 in Appendix (Figure BB.1 of the fNIRS standard) schematically shows the general design of the phantom. States A and B of the phantom correspond to the different apertures (A—larger, B—smaller) aligned with the central axes of both attenuators. For the baseline state A, it is specified that the optical loss (*OL*, definition see Sec. 6.1 in Appendix) is greater than 40 dB. Some of the tests in the fNIRS standard require an even greater *OL* in state A (>60  dB). Sixty dB *OL* can be achieved by adding a neutral density filter attached to the entrance face of a 40-dB diffusive attenuator. The standard requires Δ*OL*, the change in *OL* between states A and B, to be between 3 and 4 dB, and the diameters of both internal apertures not to exceed 8 mm.

These comprehensive specifications provide a general framework but leave options for the implementation. In particular, the material of the attenuators, their specific dimensions, and the specific size of the internal apertures are not prescribed in the standard. Further, for the optical properties, ranges are defined rather than specific values.

### 2.2 Implementation of the fNIRS Standard Phantom

#### 2.2.1 Design of the phantom prototype

**Fig. 1:**
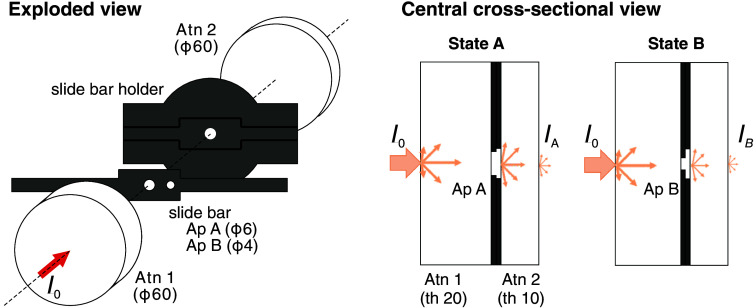
Schematic diagrams of the prototype implementing the fNIRS standard phantom. The phantom consists of two cylindrical attenuators (Atn), 1 and 2, a slide bar of 0.5-mm thickness containing two apertures (Ap) A and B with different diameters, and its holder. The distance between Atn 1 and 2 is 1.2 mm. The numbers in parentheses indicate relevant dimensions (thickness and diameters, represented by th and ϕ, respectively) of the parts of the prototype (in mm). Light input and output, as well as the fixed aperture in the holder (diameter 8 mm) and in-use switchable aperture, are co-axial.

**Fig. 2:**
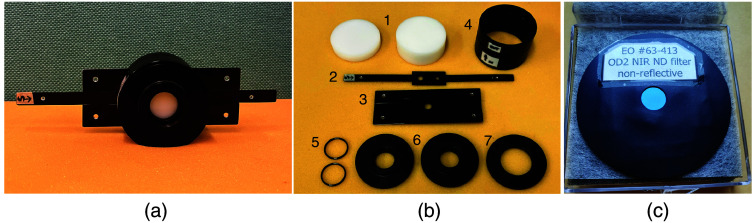
(a) fNIRS standard phantom prototype as used in both phantom campaigns. (b) Parts of the phantom prototype. 1—attenuators, 2—slider with apertures, 3—holder of slider, 4—black barrel cover, 5—C-mount rings, 6—flanges for optodes, 7—flange for power meter, and (c) filter to increase the optical loss to 60 dB.

Figure 1 shows a schematic, and Fig. 2(a) shows a photo of a prototype that implements the fNIRS standard phantom. The material for the attenuator is polyoxymethylene (POM), a white, turbid plastic material that has a reduced scattering coefficient generally comparable to that of biological tissue. In addition, the absorption coefficient is very low and nearly constant across the wavelength range of the light sources used in most fNIRS instruments.

The mechanism for changing the attenuation is realized as follows: As illustrated in Fig. 1, two holes are arranged on a long, thin bar, and the aperture size can be changed by sliding the bar between two stops. The slide bar is made of aluminium and anodized to reduce the surface reflection of near-infrared light. The two switchable apertures are 6.0 and 4.0 mm in diameter, respectively; the ratio of their areas is 2.25.

The slide bar holder has stoppers to ensure that each aperture, when in use is reproducibly centered on the optical axis. The other parts shown in Fig. 2(b) are not specified in the standard. These parts are used to hold the main parts mentioned above in place and to reduce the influence of ambient light, which is particularly important because of the low absorption of POM. Adapters for attaching the optodes of fNIRS instruments of different manufacturers and the sensor of the power meter with good positioning reproducibility are also beneficial for accurate testing.

Replicas of the fNIRS standard phantom prototype were used in both phantom measurement campaigns (2010 to 2011 in Japan, 2022 to 2023 in Europe). In the second campaign, AIST additionally provided a high-grade calibrated optical power meter type 8320 with optical sensor 82321B (ADC Corporation, Japan) to facilitate accurate characterization of the phantom and the required reference measurements.

#### 2.2.2 Selection of turbid phantom material

POM samples evaluated for this phantom were Duracon^®^ (NE25X, Polyplastics, Japan) and Polypenco^®^ Acetal (POM-NC, Polypenco, Japan), both copolymers. The bulk of each material was machined into three 40  mm×40  mm×5  mm sections with surface roughness Ra of 0.8  μm. The diffuse transmittance and diffuse reflectance of each section were measured using a spectrophotometer with an integrating sphere (UV-2600i and ISR-2600Plus for UV-2600, Shimadzu Corp., Kyoto, Japan). Prior to this experiment, one sample of Duracon and one of Polypenco Acetal were repeatedly removed and inserted in the spectrophotometer 5 times to check for repeatability of both transmittance and reflectance measurements. Based on the results of these studies (see Sec. 3.1), Duracon was selected.

#### 2.2.3 Filter to increase the optical loss to 60 dB

For some of the tests described in the standard, an attenuator with *OL* of 60 dB or higher is required. Such an attenuator can be created by equipping the original phantom with an additional optical filter of an optical density of 2 or greater over the spectral range of interest, corresponding to a filter transmission ≤1%. The following optical filters were tested for their suitability to achieve this goal, (i) a 3-mm-thick volume absorptive neutral density glass filter NG3 (Schott, Germany); (ii) a reflective neutral density filter ND520B (Thorlabs, New Jersey, USA), i.e., a glass substrate with a highly reflective metal (Inconel^®^) coating on one side; and (iii) a non-reflective neutral density filter OD 2.0 NIR #63-413 [Edmund Optics, New Jersey, USA, Fig. 2(c)], i.e., a glass substrate with coatings on both sides, with reflection ≤2%.

The filter-glass–based NG3 filter had a transmission between 1% and 1.5% between 725 and 900 nm (manufacturer specifications), but only 0.3% at 670 nm and less for shorter wavelengths. It approximately fulfills the requirement of an optical density of 2 or greater; however, the resulting optical density of the phantom combined with this NG3 filter exhibits a remarkable wavelength dependence. A reflective ND filter such as the ND520B is not suitable for this purpose either: when placed close to a highly scattering material, a substantial fraction of the diffused light exiting the phantom gets reflected back into the phantom. Tests with this filter attached to the entrance face of the fNIRS phantom showed an excess in the overall transmission of the filter-phantom combination compared with the value expected from the filter transmission by about 40% (data not shown). The ND filter with minimized reflection OD 2.0 NIR #63-413 exhibited a relatively flat transmission spectrum (monotonously decreasing from 1.28% at 600 nm to 0.86% at 845 nm; own measurements). It turned out to be the best option for a wide range of wavelengths and was attached to the entrance face of the fNIRS standard phantom to achieve an overall *OL* of ∼60  dB.

### 2.3 Characterization of the Phantom Prototype: Optical Loss and Its Change

The quantity *OL* is used here as defined in the standard IEC 80601-2-71, as a property of the fNIRS standard phantom, see Sec. 6.2 in Appendix. *OL* expressed in dB can be written as $\mathrm{OL}=-10\;\log_{10}(P_{\mathrm{ex},\text{total}}/P_{\text{in}})$, where $P_{\text{in}}$ is the input power and $P_{\mathrm{ex},\text{total}}$ is the total power of light that exits a circular aperture of area $S_{\mathrm{ex}}$ as specified in the standard (diameter 8 mm, attached to and centered on the phantom’s exit face) in any direction. It should be noted that *OL* is related to T, the diffuse transmittance at the center of the phantom exit face according to $\mathrm{OL}=-10\;\log_{10}(S_{\mathrm{ex}}T)$ provided the diffuse transmittance is approximately constant over the area of the exit aperture.^28^

The characterization of *OL* of the phantom prototype as well as of its change ΔOL upon switching the internal aperture was based on the following measurements. A supercontinuum laser SuperK Fianium FIU-15 PP (NKT Photonics, Germany) equipped with a tuneable filter VARIA (NKT Photonics, Germany) was employed as a tuneable light source in the range from 600 to 845 nm with a spectral bandwidth of 10 nm and an average output power on the order of 10 mW at a repetition rate of 78 MHz. It should be noted that the pulsed character of the light source was not relevant for this measurement. A collimated beam of a diameter less than 5 mm was directed to the center of the entrance face of the phantom. The light exiting the phantom through a centered aperture of diameter 8.02 mm directly attached to its exit face was recorded by an optical power meter type 8320 with optical sensor 82321B (ADC Corporation, Japan). *OL* and ΔOL were measured as a function of wavelength in steps of 50 nm.

For *OL*, first, the ratio was obtained of the exiting power in state A to the input power. The input power was measured with the same power meter before and after each set of measurements. Before *OL* was calculated, a correction formula [Eq. (6) in Ref. 28] based on the radiant power transfer equation^29^ was applied to account for the actual size of the photosensitive area of the power meter (diameter 8.5 mm) and its finite distance from the phantom surface.

For the change ΔOL, the exiting power was recorded by the same power meter while switching the internal apertures between both positions by moving the slider manually. This procedure was repeated several times to suppress the influence of a possible drift in laser power.

### 2.4 Performance Test for fNIRS Signal Magnitude by Means of the fNIRS Standard Phantom

The present work focuses on the main application of the phantom, i.e., the test for “pathlength-dependent hemoglobin change,” which stands for the fNIRS signals representing the product of mean optical pathlength and the concentration changes of oxyhemoglobin ($O_{2}\mathrm{Hb}$) and deoxyhemoglobin (HHb), respectively (see Sec. 6.1 in Appendix).

The test procedure follows the description provided in Annex BB 3.1 of the standard IEC 80601-2-71:2025 (see Sec. 6.3 in Appendix). The test consists of the main measurement with the device under test, a reference measurement, and their comparison:

• The main measurement aims at estimating the pathlength-dependent oxy- and deoxyhemoglobin changes ($\Delta c_{O2\mathrm{Hb}}L$ and $\Delta c_{\mathrm{HHb}}L$) that the fNIRS device under test reports when the phantom is switched from state A (baseline state) to state B by changing the size of the internal aperture. The emitter optode is attached to the entrance side, the detector optode to the exit side of the phantom. The data acquisition protocol is identical to that for *in vivo* measurements.
• The reference measurement relies on an independent estimation of the attenuation change between states A and B of the phantom at all nominal wavelengths of the fNIRS device under test. The light source can be an external stabilized light source or the source optode of the device under test. The power exiting the phantom is recorded by a sufficiently sensitive, calibrated power meter. The attenuation change ΔA occurring between states A and B is calculated for each nominal wavelength by applying Eq. (2) (see Sec. 6.3 in Appendix), where the light intensities $I_{A}$ and $I_{B}$ are replaced with the related measured power values. The corresponding reference values ${\left(\Delta c_{O2\mathrm{Hb}}L\right)}_{\mathrm{Ref}}$ and $(\Delta c_{\mathrm{HHb}}L{)}_{\mathrm{Ref}}$ are calculated by applying Eqs. (4) and (5) (see Sec. 6.3 in Appendix). These formulas are valid for the two-wavelength case. If the fNIRS device has more than two wavelengths, the linear system of equations in Eq. (3) can be solved as well.
• The results obtained for $\Delta c_{O2\mathrm{Hb}}L$ and $\Delta c_{\mathrm{HHb}}L$, respectively, from the main and the reference measurements are compared. The fNIRS standard requires the relative differences to be less than 5% for $O_{2}\mathrm{Hb}$ as well as for HHb.

It should be noted that the results for $\Delta c_{O2\mathrm{Hb}}L$ and $\Delta c_{\mathrm{HHb}}L$ from this phantom test cannot be compared between different fNIRS devices. These results depend on the set of wavelengths used, even if the attenuation change ΔA is the same and independent of wavelength. For more details, see Sec. 4.3.

## 3 Results

### 3.1 Selection of the Phantom Material

**Fig. 3:**
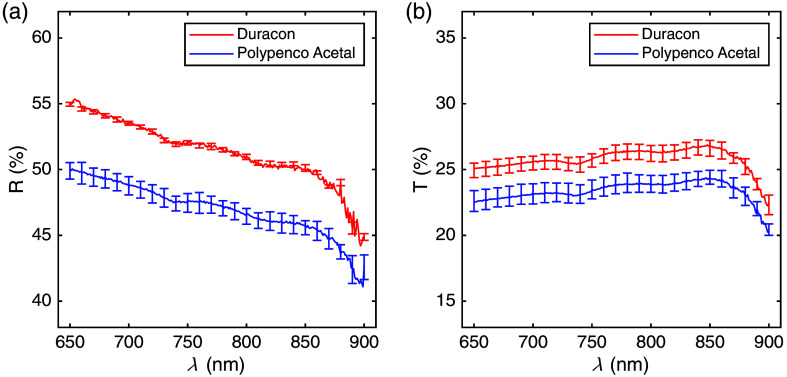
Diffuse reflectance (R) and diffuse transmittance (T) of POM materials, Duracon and Polypenco Acetal as a function of wavelength (λ). The average from three samples (40  mm×40  mm×5  mm sections) for each material is shown as solid lines. The upper and lower limits of the error bars indicate the maximum and minimum values among the samples, respectively.

The diffuse reflectance and transmittance of sections of Duracon and Polypenco Acetal are shown in Fig. 3. The wavelength dependence of diffuse reflectance and transmittance was similar for both materials, but Duracon had higher values than Polypenco Acetal. The diffuse reflectance fell off at longer wavelengths and decreased more markedly at wavelengths above 850 nm. The diffuse transmittance tended to be higher with increasing wavelength in the wavelength range from 650 to 850 nm and decreased with increasing wavelength above 850 nm. Between 650 and 900 nm, the standard deviations of diffuse reflectance (0.16% versus 0.56%) and transmittance (0.48% versus 0.58%) among samples were smaller in Duracon than in Polypenco Acetal. Based on these results, Duracon was selected as the material for the phantom prototype.

### 3.2 Optical Loss and Its Change

**Fig. 4:**
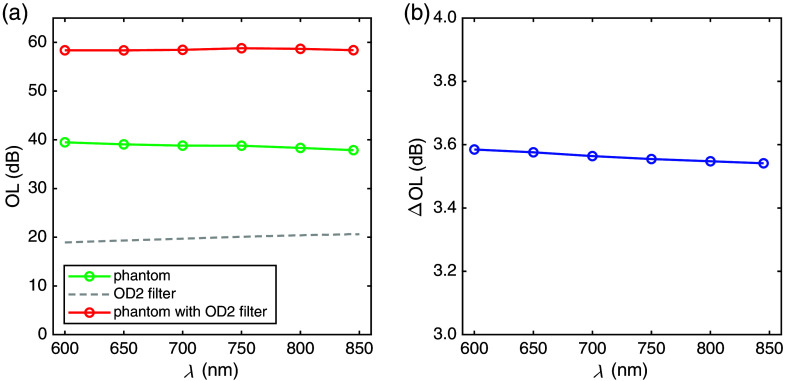
(a) Optical loss (*OL*) as a function of wavelength (λ) for the phantom prototype in state A, with and without a neutral density filter with low reflectivity (OD 2.0 NIR #63-413, Edmund Optics) attached to its entrance face, together with the filter spectrum. (b) Change in the optical loss (ΔOL) of the phantom prototype due to a change in the diameter of the internal aperture from state A to state B. The estimated uncertainty is 0.04 dB.

Figure 4(a) presents *OL* of the fNIRS phantom prototype with and without a neutral density filter of nominal optical density 2 (transmission 1%, attenuation 20 dB). The results refer to measurements with the required aperture of 8 mm diameter attached to the exit face of the phantom. In both cases, the requirements to achieve an optical loss of 40 and 60 dB, respectively, were met rather well. The values measured were slightly less than the required values; however, the difference does not substantially exceed the estimated overall uncertainty of 1 dB.

The phantom exhibits a minor wavelength dependence in *OL* (1.6 dB decrease from 650 to 845 nm). In the 60-dB case, the wavelength dependence is even less because the spectrum of the added specific OD2 filter exhibits a slight decrease in transmission for increasing wavelengths, corresponding to an increase in attenuation by 1.7 dB, as derived from the transmission data of the chosen filter given in Sec. 2.2.3, over the wavelength range plotted in Fig. 4(a). Incidentally, this increase nearly completely compensates for the decreasing *OL* of the phantom itself.

The uncertainty in the estimation of *OL* includes several factors. Perfect linearity of the power meter over six decades is challenging (example: 10 mW input corresponds to outputs of 1  μW and 10 nW for 40 and 60 dB, respectively). Several parameters necessary for the radiometric correction of the recorded power (effective position, diameter, and homogeneity of the photosensitive area of the power meter) are not exactly known. The overall uncertainty was estimated as 1 dB, corresponding to a 20% relative uncertainty of the power ratio. Error bars are not shown in Fig. 4(a), as they would barely exceed the size of the symbols.

Figure 4(b) shows the measured change ΔOL of the phantom prototype when switching from the larger (state A) to the smaller (state B) internal aperture. The change ΔOL is well within the required range from 3 to 4 dB. Based on the specific phantom construction, the expected change is 3.52 dB, as given by the ratio of the areas of the internal apertures (2.25). The ΔOL values plotted in Fig. 4(b) are slightly larger than the expected value and exhibit a minor decrease with wavelength within the range investigated, from 3.58 dB at 600 nm to 3.54 dB at 845 nm, corresponding to a decrease in the power ratio by 0.9%.

### 3.3 Application of the Phantom Prototype for Testing fNIRS Devices

**Fig. 5:**
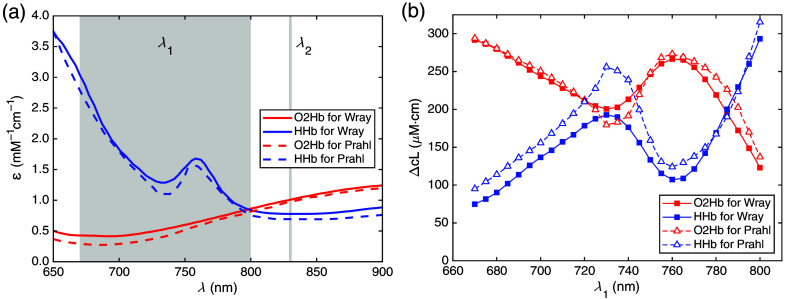
Wavelength dependence of pathlength-dependent hemoglobin changes as measured on the fNIRS standard phantom. (a) Molar decadic absorption coefficients ε for HHb and $O_{2}\mathrm{Hb}$ based on Wray et al.^30^ and Prahl.^31^ (b) Pathlength-dependent hemoglobin changes calculated as a function of $\lambda_{1}$ in the range from 670 to 800 nm with fixed $\lambda_{2}=830\;\mathrm{nm}$ and constant ΔA=0.3522, for both sets of absorption spectra plotted in (a) with $\lambda_{1}$ and $\lambda_{2}$ highlighted in gray.

The phantom prototype presented in Sec. 2.2.1 (see Figs. 1 and 2) has been applied for performance testing of fNIRS devices in two phantom campaigns. In the first phantom campaign, pathlength-dependent hemoglobin changes were measured on the replicas of the phantom prototype with commercial fNIRS devices of three Japanese manufacturers. In the second phantom campaign (2022 to 2023), utilizing one of the phantoms from the original 2010 campaign, two European manufacturers performed the test procedure for pathlength-dependent hemoglobin changes with their devices (see Table 1). The values for $\Delta c_{O2\mathrm{Hb}}L$ and $\Delta c_{\mathrm{HHb}}L$ measured for all devices were of similar magnitude, but at least in part clearly different from device to device. It is important to note that these data do not indicate the relative superiority or inferiority of devices: The results depend on the specific set of wavelengths of each device, and a comparison between devices is not meaningful. In addition, the set of absorption spectra adopted has a considerable influence on the results. The results for device VI exhibit a different order than those of the other devices, i.e., $\Delta c_{\mathrm{HHb}}L>\Delta c_{O2\mathrm{Hb}}L$. However, this finding is entirely plausible for the wavelengths of this device, together with a specific set of spectra that differs from the sets that were used as examples in Fig. 5. It should be noted that information about the set of absorption spectra implemented in the various devices was not available from the manufacturers. For further explanations on the performance test for pathlength-dependent hemoglobin changes, refer to the discussion in Sec. 4.3.

**Table 1:** Pathlength-dependent hemoglobin changes measured on the fNIRS standard phantom prototype by various devices from Japanese manufacturers (Hamamatsu Photonics K.K, Hitachi Ltd, Shimadzu Corp) and European manufacturers (Artinis Medical Systems BV, Netherlands, and NIRx Medizintechnik GmbH, Germany). The device names were anonymized. Note that one manufacturer performed the measurements with two different devices.

Device	I	II	III	IV	V	VI
Wavelengths (nm)	695, 830	780, 805, 830	775, 810, 850	760, 845	760, 850	785, 830
$\Delta c_{O2\mathrm{Hb}}L$ (μM cm)	243.6	230.9	242.9	222.8	215.3	166.8
$\Delta c_{\mathrm{HHb}}L$ (μM cm)	124.3	159.1	154.5	133.8	133.7	224.9

The related measurements of the pathlength-dependent hemoglobin changes were successfully completed with all devices. For each device under test, the values obtained from the device output and from the associated reference measurement (with the same set of wavelengths) agreed within 5% as required in the standard, for both, $\Delta c_{O2\mathrm{Hb}}L$ and $\Delta c_{\mathrm{HHb}}L$.

## 4 Discussion

### 4.1 Implementation of the fNIRS Standard Phantom

An fNIRS standard phantom is required to be suitable for performing the tests specified in the standard. The most relevant characteristics of this phantom are a specified total diffuse attenuation (or *OL*) and a method to change it by a well-defined amount. In addition, the phantom should be device agnostic and usable for testing of devices under industrial conditions.

Diffuse optics research has primarily utilized phantoms for the development of novel measurement techniques and system checks of the devices.^32^ Here, phantoms are often required to mimic the optical properties of biological tissues. This can be achieved by independently controlling scattering and absorption properties by adding appropriate substances to a base material such as epoxy resin or silicone. However, in case of the fNIRS standard phantom such closeness to tissue optical properties is not required, and any type of turbid material can be considered. In the wavelength range from 650 to 850 nm, the scattering coefficient of white POM is high and generally comparable to that of biological tissue, whereas the absorption coefficient is approximately constant and considerably lower than that of biological tissues. The constant absorption coefficient is extremely useful for testing various devices equally using the same phantom. In addition, white POM is readily available in solid bulk and is easily machinable. These features make white POM a good choice for the attenuator material of the fNIRS standard phantom. Note that the standard also allows the fabrication of attenuators with epoxy or silicon resins; however, careful consideration must be given to the influence of the absorption properties of the base material on the characteristics of the phantom (*OL*, ΔOL) and their wavelength dependence. These aspects will be tackled in the companion paper.^27^

There is a wide variety of white POM bulk products available, and their properties can vary due to differences in molecular chains for chemical stability (copolymer or homopolymer), and moulding methods, all of which may affect optical homogeneity. In particular, “center line porosity” is a known issue in POM-H such as Delrin. In this work, homogeneity was evaluated by measuring diffuse reflectance and transmittance of several sections of samples of two different POM-C (copolymer) materials (Fig. 3) machined to surface roughness Ra=0.8  μm. The more homogeneous material of these two samples, Duracon, was selected for the phantom prototype.

The basic optical principle of the phantom prototype is consistent with the general concept as proposed earlier by Firbank and Delpy.^26^ This concept includes apertures of variable diameter sandwiched between two turbid blocks. A largely wavelength-independent attenuation change is achieved by switching between different apertures. However, the design of the present phantom prototype is less complex and more appropriate for performing the standard tests. The major difference is the reduction to two internal apertures to create a single attenuation change, whereas Firbank’s design features eight apertures. A related advantage of the present design is the reduction of the total thickness of the slider and its holder from 13.5 mm in Firbank’s design to 1.2 mm. This modification reduces the light attenuation by the slider aperture walls and simplifies the estimation of ΔOL, which solely depends on the diameter of the apertures. For the present phantom prototype, a well-defined, appropriate total optical loss was specified (OL>40  dB). By inserting a specified aperture (8 mm) on the exit face and defining *OL* via the ratio of the total optical power exiting this aperture to the input power, the diffuse attenuation of the composite phantom can be assessed in a simple, standardized way. Another difference between the two phantoms lies in their materials. The phantom prototype uses commercially available POM as its turbid material, which is advantageous because it does not require the manufacturing of a homogeneous solid turbid medium with strict formulation of additives to adjust the absorption and scattering coefficients.

Various groups have developed different types of fNIRS phantoms devoted to performance testing of specific aspects of fNIRS instrumentation and data analysis methods. A major aspect is the quantitative simulation of localized absorption changes that occur in the brain due to changes in oxy- and deoxyhemoglobin concentrations. A frequent approach is the use of layered phantoms to assess the depth selectivity of fNIRS measurements. Jelzow et al. used a two-layer liquid phantom with a stepwise increase of the absorption coefficient in each layer to test a depth-selective reconstruction method for time-domain fNIRS measurements.^33^ Funane et al. constructed an all-solid phantom with a POM base containing two layers at different depths, in which turbid slabs with higher absorption could be moved in and out of the region of interest.^34^ Kawaguchi et al. developed a more realistic phantom with four solid layers where the optical properties of two of them mimicking scalp and brain could be changed by moving different parts independently.^35^ These solid layered phantoms were used to validate analysis methods employing measurements at different source-detector separations to eliminate the influence of superficial hemoglobin changes on CW fNIRS signals.

Phantoms that more accurately replicate the anatomical structure of the human head and localized absorption changes have also been developed. Tanikawa et al. fabricated a phantom that precisely modeled the anatomical structure of the human head by integrating magnetic resonance imaging data and stereolithography.^36^ In addition, they designed a system to induce localized absorption changes by switching the liquid circulation pathway.^37^ Barbour et al. developed the sophisticated head phantom equipped with programmable electrochromic cells capable of arbitrarily modulating local absorptions over time.^38^ These techniques enable the construction of phantoms that closely emulate both the anatomical and functional properties of the human brain, providing valuable tools for the validation of advanced functional neuroimaging methods such as diffuse optical tomography.

A particularly simple and effective method to mimic localized absorption changes (in both depth and lateral direction) was developed by Martelli et al.,^39^^,^^40^ showing that a totally absorbing object of a certain (small) volume yields virtually the same attenuation change that would be measured for a realistic absorption change within a larger volume.^39^ Such phantoms were first implemented with black polyvinyl chloride cylinders suspended in a liquid phantom based on Intralipid and ink^40^ and used in a multi-laboratory campaign devoted to performance assessment of instruments for time-domain fNIRS imaging, as part of the European project “nEUROPt.”^41^ A solid implementation of this type of phantom was published by Pifferi et al.^42^ and used in the BitMap campaign.^43^

These phantoms with black inclusions have the potential utility in performance tests required in future versions of the fNIRS standard including FD and TD fNIRS techniques. In the context of the present work, it is interesting to consider whether phantoms with black inclusions would be suitable for testing in CW fNIRS as well. The current phantom is ill-suited for reflection geometry; however, phantoms with inclusions may be used in either reflection or transmission geometry. On the contrary, the attenuation change introduced by the presence of the black inclusion is not a distinct value but depends on the emitter-detector separation and on the scattering and absorption coefficients of the surrounding turbid medium. Thus, the attenuation change cannot be estimated in a straightforward manner but requires dedicated Monte-Carlo simulations. Moreover, the attenuation change is generally wavelength-dependent, specifically due to the inevitable wavelength dependence of the reduced scattering coefficient.

As a result of these considerations, the advantages of the method used with the fNIRS standard phantom to create an attenuation change become obvious. The change in attenuation (equivalent to ΔOL) is (i) essentially given by the ratio of the areas of the changeable internal apertures and (ii) therefore essentially independent of wavelength. This feature is a firm basis for various tests performed with the fNIRS standard phantom, including tests for signal-to-noise ratios. All wavelengths occurring in fNIRS devices are treated equally, avoiding any preference for specific sets of wavelengths that may differ from device to device.

### 4.2 Optical Loss and Its Changes

The results for *OL* of the fNIRS standard phantom prototype [see Fig. 4(a)] show that this implementation meets the requirements of the standard well, both for the 40 dB and the 60 dB case. The values of *OL* do not quantitatively influence the results of any of the tests in the standard directly. However, the high *OL* ensures that the light propagation within the phantom is diffuse, and the power as well as the angular distribution of the light exiting the phantom are similar to those of light exiting the human head in fNIRS measurements.

It should be noted that *OL* is not a unique characteristic of the composite phantom itself. Its value critically depends on the diameter of the exit aperture (specified as 8 mm) that limits the area of the radiating surface “seen” by the power meter. Moreover, apart from the size of the exit aperture, the power recorded depends on the finite distance of the photosensitive area of the power meter from the phantom surface and its actual diameter. To eliminate these influences on the estimation of *OL*, a radiometric correction is included in the measurement.^28^

The actual total light attenuation experienced by an fNIRS device can differ from *OL*. The amount of light that hits its detector optode depends on its effective receiving area, which can be different from the area of the 8-mm–diameter exit aperture, and on its acceptance angle. As opposed to *OL*, the ΔOL measurement is comparatively robust against geometric conditions because only the ratio (∼2) of two power values is relevant. Here, the radiometric correction is not necessary and the size of the area on the exit face from which the light is collected has a very minor influence.

The *OL* of the phantom is mainly achieved by (i) strong scattering and (ii) the internal aperture. In POM scattering, and thus *OL*, decreases with wavelength. The minor, but reproducible, wavelength dependence of ΔOL is most likely due to the finite size of the apertures relative to the scattering-dependent spatial light distribution inside the attenuators. More insights into light propagation within the fNIRS standard phantom are provided in the companion paper.^27^

Switching an internal aperture is an effective method to achieve an almost completely wavelength-independent ΔOL and thus attenuation change ΔA, as already mentioned in Firbank’s work.^26^ However, ignoring residual wavelength dependence when calculating $(\Delta c_{O2\mathrm{Hb}}L{)}_{\mathrm{Ref}}$ and $(\Delta c_{\mathrm{HHb}}L{)}_{\mathrm{Ref}}$ can cause deviations of up to 2% (device output and reference values are required to be within <5%). Therefore, it is recommended to measure ΔA for each wavelength of the device under test accurately and to include the wavelength dependence ΔA(λ) in Eqs. (4) and (5) (Sec. 6.3 in Appendix) when calculating the reference values.

It is interesting to discuss whether a switchable broadband filter in front of or within a diffuse attenuator could be an alternative to creating ΔOL by switchable internal apertures. However, it might not be trivial to achieve an attenuation spectrum of a (non-reflecting) filter that is similarly flat as the ΔOL spectrum of the prototype, in a robust and reproducible manner.

Furthermore, it is worth noting that this work only assesses the phantom at wavelengths less than 850 nm, as are typically used in commercial fNIRS devices. Beyond that limit, the flatness of the *OL* and ΔOL spectra is not necessarily preserved. Additional measurements of *OL* and ΔOL up to 900 nm (not shown here) reveal only a moderate increase of *OL* to 45 dB at 900 nm, where POM exhibits a major absorption peak. This finding is qualitatively consistent with the decrease in diffuse transmittance of a thinner sample of the material toward 900 nm (see Fig. 3). The POM absorption peak also influences ΔOL, but to a very minor extent, a decrease by <0.06  dB compared with 850 nm.

### 4.3 Performance Test for Pathlength-Dependent Hemoglobin Change

It is important to realize that the results for the signal magnitude, i.e., pathlength-dependent hemoglobin change ($\Delta c_{O2\mathrm{Hb}}L$ and $\Delta c_{\mathrm{HHb}}L$), measured on the fNIRS standard phantom cannot be compared between devices utilizing different wavelengths, as these results are wavelength dependent. This dependence is illustrated by calculations displayed in Fig. 5 for an fNIRS device with two wavelengths $\lambda_{1}$ and $\lambda_{2}$. Figure 5(a) shows the absorption spectra of $O_{2}\mathrm{Hb}$ and HHb used, i.e., two sets of spectra from different literature sources.^30^^,^^31^ Furthermore, a constant attenuation change ΔA=0.3522, corresponding to the nominal ΔOL of the fNIRS standard phantom prototype was assumed. The signals $\Delta c_{O2\mathrm{Hb}}L$ and $\Delta c_{\mathrm{HHb}}L$ were calculated based on Eqs. (4) and (5), with $\lambda_{1}$ (shorter wavelength) and fixed $\lambda_{2}$ [see Fig. 5(b)]. The shorter wavelength $\lambda_{1}$ was varied from 670 to 800 nm, where the HHb spectra exhibit pronounced changes, whereas the longer wavelength was kept fixed at $\lambda_{2}=830\;\mathrm{nm}$.

The calculated results of the phantom-based tests described here, the magnitudes of both $\Delta c_{O2\mathrm{Hb}}L$ and $\Delta c_{\mathrm{HHb}}L$, vary widely and non-monotonically with $\lambda_{1}$. For example, for $\lambda_{1}=670\;\mathrm{nm}$, $\Delta c_{O2\mathrm{Hb}}L$ is about 4 times larger than $\Delta c_{\mathrm{HHb}}L$, whereas both signals are nearly equal when the shorter wavelength is $\lambda_{1}=730\;\mathrm{nm}$, in case the Wray spectra are taken. The dip of the HHb spectrum at 730 nm and the peak at 760 nm are reflected in both signals. It should be noted that the strong wavelength dependence is not due to the phantom, where ΔA is virtually wavelength independent [Fig. 4(b)], but rather due to the absorption spectra $\varepsilon_{O2\mathrm{Hb}}(\lambda )$ and $\varepsilon_{\mathrm{HHb}}(\lambda )$ used in the analysis. Conversely, in a real *in vivo* fNIRS measurement, ΔA(λ) carries the full spectral information according to Eq. (3) in Sec. 6.3 in Appendix. In this case, solving this system of equations via Eqs. (4) and (5) yields the actual hemoglobin changes, independently of the set of wavelengths used in the measurement.

The calculations also represent an example of the considerable influence of the choice of absorption spectra from various literature sources. Figure 5(a) shows the clearly different spectra from the two publications selected here. Similar differences are found between the spectra of other authors; for a comparison of multiple spectral data sets for hemoglobin, see the literature.^44^ The reason for this diversity seems to be the difficulty in preparing completely oxygenated and deoxygenated hemoglobin for spectral measurements. The consequences for the magnitudes of both $\Delta c_{O2\mathrm{Hb}}L$ and $\Delta c_{\mathrm{HHb}}L$ are substantial. As an example, for $\lambda_{1}=730\;\mathrm{nm}$, where application of the Wray spectra yields nearly equal values, $\Delta c_{O2\mathrm{Hb}}L$ exceeds $\Delta c_{\mathrm{HHb}}L$ by more than 30% with the Prahl spectra. Hence, for the phantom test of fNIRS signal magnitudes to succeed, it is relevant to use the same absorption spectra in the calculation of the reference values as implemented in the device algorithm. Likewise, the use of different absorption spectra can also lead to quantitative differences between *in vivo* fNIRS measurements performed with different devices.

Having in mind a substantial wavelength dependence as shown in Fig. 5(a), it is obvious that there are no unique target values for $\Delta c_{O2\mathrm{Hb}}L$ and $\Delta c_{\mathrm{HHb}}L$ resulting from the performance test on the phantom. Hence, the comparison between the measurements on a device under test and the related reference measurements (see Sec. 2.4) with the same set of (device-specific) wavelengths is the only comparison that is required in the fNIRS standard.

One relevant aspect of this comparison is a check of the device’s analysis algorithm. The calculation in case of the reference measurement is prescribed in the standard and entirely transparent, whereas the algorithm implemented in the commercial fNIRS device under test is not necessarily known to someone performing the test. Failure of this test can be caused by (i) performing the reference calculation with other wavelengths than those implemented in the device algorithm and (ii) the use of different sets of hemoglobin absorption spectra in both calculations. Yet, if the device under test has actual wavelengths deviating from the nominal values assumed in both calculations equally, this cannot be detected by the test since ΔA generated by the phantom is virtually independent of wavelength.

Moreover, a common pitfall in fNIRS is confusion of decadic and natural logarithm ($\log_{10}$, $\log_{e}$) in the calculation of ΔA. In case the device algorithm uses decadic ε spectra but defines ΔA via $\log_{e}$ and the reference calculation of $\Delta c_{O2\mathrm{Hb}}L$ and $\Delta c_{\mathrm{HHb}}L$ follows the way required in the standard ($\log_{10}$), the results of the phantom test would exhibit a clear discrepancy by a factor of 2.3. Conversely, a successful comparison, i.e., a relative difference of <5% between measurement by the device under test and reference measurement, can rule out such an error in the device’s analysis algorithm.

The test for fNIRS signal magnitude relies on a single attenuation change only, i.e., linearity is not explicitly addressed. Nevertheless, a successful comparison requires the measurement of ΔA by the device under test to deliver a value that is close to ΔA recorded in the reference measurement. A major nonlinearity in the relevant operating range of the device, when recording the ratio of two power values that are about a factor of 2 apart, could cause the test to fail.

It should be noted that the magnitudes of $\Delta c_{O2\mathrm{Hb}}L$ and $\Delta c_{\mathrm{HHb}}L$ obtained in this test are not comparable with typical fNIRS signals. The latter are of the order of 20  μM⋅cm, i.e., around a factor of 10 smaller. Moreover, for a typical brain activation, $\Delta c_{O2\mathrm{Hb}}L$ is positive, i.e., $c_{O2\mathrm{Hb}}L$ is larger than the baseline state, and $\Delta c_{\mathrm{HHb}}L$ is negative, whereas the respective results of the phantom test are both positive. To address small signals, the fNIRS standard requires additional performance tests that are related to signal-to-noise ratios of several quantities, a topic beyond the scope of the present publication.

It is worth discussing the 5% limit of the relative difference between the measurement on a device under test and the associated reference measurement in the context of fNIRS accuracy. In clinical applications of fNIRS, which are addressed with the medical device standard IEC 80601-2-71, the absolute accuracy of the hemoglobin concentration changes rarely matters, whereas the temporal profile of these signals is often the relevant result. The magnitude of fNIRS signals measured on the human head critically depends on the thickness of extracerebral layers (skin, skull), the overlap of the sampled region and the activated region of the cortex (partial volume effect), as well as source-detector separation and interference of superficial signals. These influences are mostly large compared with 5% of the signal magnitude.

### 4.4 Limits of Application of the fNIRS Standard Phantom

One disadvantage of the design of the fNIRS standard phantom is the challenge in utilizing this phantom for reflectance probes with inseparable emitter and detector optodes. For the next revision of the standard, a solution should be found for a reflectance-type phantom with similar attenuation characteristics.

It should be noted that the standard IEC 80601-2-71 as well as the fNIRS standard phantom are applicable to CW fNIRS equipment only. Recent advances in frequency-domain and time-domain fNIRS technologies promise their successful clinical application. Related dedicated reliable, robust, and easy-to-use phantoms for standardized performance tests need to be developed that address these advanced technologies, including their depth-selective capabilities.

## 5 Conclusions

The fNIRS standard phantom described and specified in the standard IEC 80601-2-71 for fNIRS equipment is the major basis for performance testing in this standard. It relies on two turbid blocks and two switchable circular apertures in between them that are used to create a reproducible change in diffuse attenuation. We present a phantom prototype designed and manufactured in Japan that implements this fNIRS standard phantom in a way that is easy to replicate and robust in use. The characterization of this phantom prototype in terms of OL(λ) and ΔOL(λ) demonstrated that this prototype meets the specifications in the standard. A specific useful finding was the very minor wavelength dependence in ΔOL.

The usability of the phantom prototype was assessed by various fNIRS manufacturers conducting the main performance test in IEC 80601-2-71 in two measurement campaigns, in Japan and Europe. They were able to perform this test successfully, showing that the function of the prototype fulfils the requirements. Meanwhile, replicas of the prototype became commercially available from a phantom manufacturer (BioPixS Ltd., Cork, Ireland), which is another major outcome of the second campaign.

The practical work with the phantom prototype as well as the performance testing provided a basis for a deeper understanding of both. This experience is represented in the extended discussion in this publication. The companion publication,^27^ another result of the activities of European research groups during the measurement campaign on the phantom prototype, goes a step further. It covers the spectral characterization of the scattering and absorption properties of the turbid material used in the prototype and the subsequent modelling of the characteristics of the composite phantom by Monte-Carlo simulations. The detailed understanding of the function of this fNIRS standard phantom will facilitate the future development of standardization-related advanced phantoms for fNIRS and similar optical methods.

This work can become a useful resource for fNIRS manufacturers and providers of phantoms for biomedical optics. It will foster the necessary inclusion of standardization in the development of clinically relevant fNIRS devices and methods.

## 6 Appendix

### 6.1 Relevant terms and definitions from IEC 80601-2-71:2025

The terms and definitions in IEC80601-2-71 relevant to this paper are listed below with the standard and subclause numbers given in parentheses. Due to the request of the Japanese local standard association, the terms and definitions are given in the exact form in which they appear in the standard, i.e., terms in the standard are capitalized, and they appear in the appendices of this paper as well. Terms not listed here can be found in the IEC 60601-1 series including IEC 80601-2-71. Note that ME EQUIPMENT indicates fNIRS equipment hereafter, and the exact definition can be found in IEC 60601-1:2005+AMD1:2012 +AMD2:2020, subclause 3.63.

**FUNCTIONAL NIRS PHANTOM**apparatus that simulates a PATHLENGTH-DEPENDENT HEMOGLOBIN CHANGE by giving the ME EQUIPMENT a specified known change in attenuation to evaluate the difference between the measured value of the pseudo PATHLENGTH-DEPENDENT HEMOGLOBIN CHANGE obtained from the measurement on the phantom and the reference value calculated from the attenuation change

(IEC 80601-2-71:2025, 201.3.208)

**OPTICAL LOSS**ratio of the total optical power exiting the FUNCTIONAL NIRS PHANTOM or attenuator through a specified aperture, to the optical power emitted by the EMITTER OPTODE connected to the FUNCTIONAL NIRS MONITOR and placed on the entrance side of the FUNCTIONAL NIRS PHANTOM or attenuator

(IEC 80601-2-71:2025, 201.3.210)

**PATHLENGTH-DEPENDENT HEMOGLOBIN CHANGE**

Δc·Lcollective term which signifies the product of apparent hemoglobin concentration change and the mean optical pathlength inclusive of two chromophores (oxyhemoglobin and deoxyhemoglobin), as well as total hemoglobin change

(IEC 80601-2-71:2015, 201.3.212)

### 6.2 Brief Description of FUNCTIONAL NIRS PHANTOM based on IEC 80601-2-71:2025

**Fig. 6:**
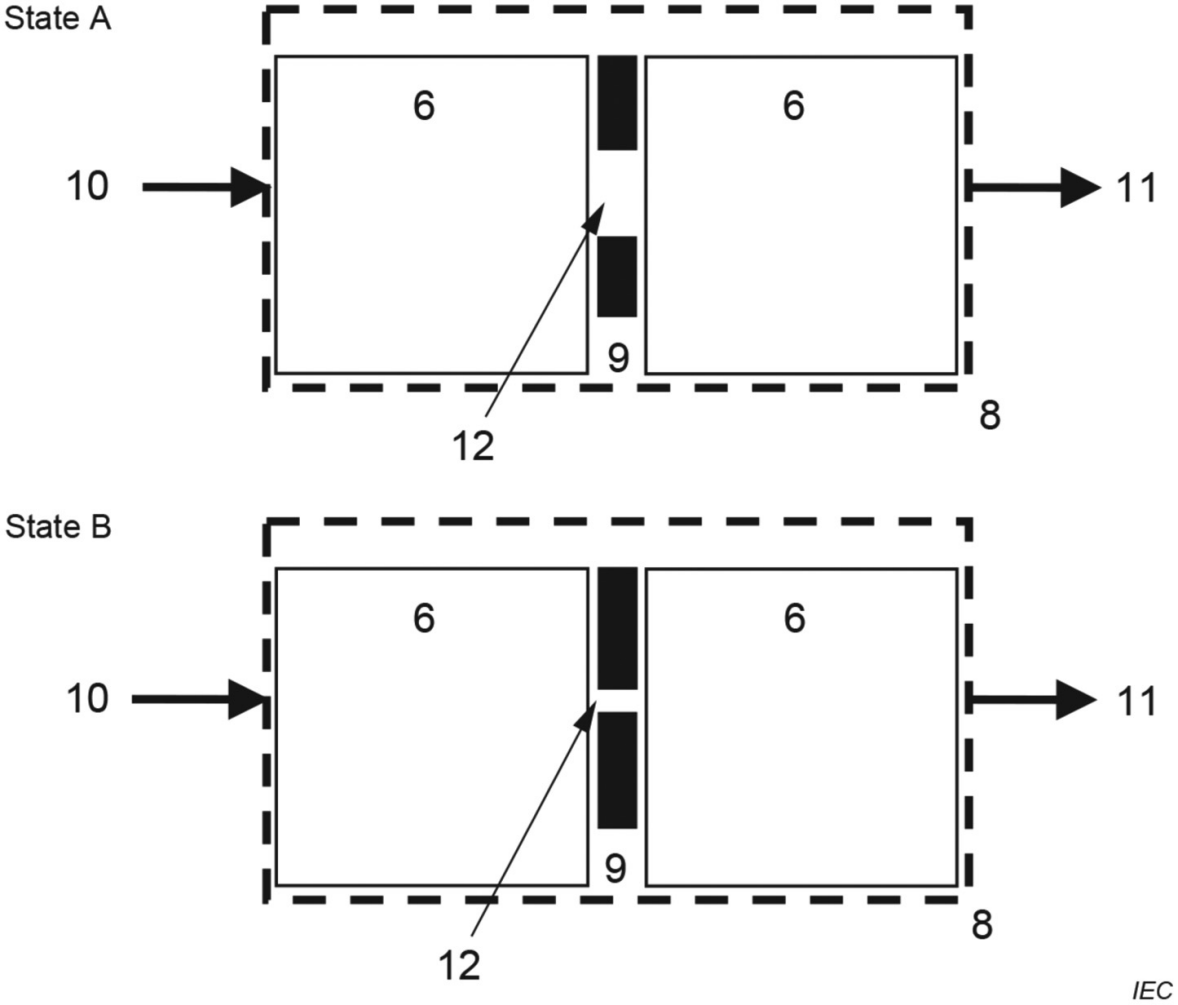
The FUNCTIONAL NIRS PHANTOM in two states with different detected light intensities (Figure BB.1 in IEC 80601-2-71:2025, reprinted with permission). The numbering is in accordance with the standard and means the following: six Attenuator, eight FUNCTIONAL NIRS PHANTOM, nine changeable aperture, 10 Incident light intensity l0, 11 detected light intensity: $I_{A}$ and $I_{B}$, respectively, and 12 area of aperture.

FUNCTIONAL NIRS PHANTOM should provide two light intensities $I_{A}$ and $I_{B}$ in states A and B. Figure 6 (Figure BB.1 in the standard) shows the schematic diagram of the FUNCTIONAL NIRS PHANTOM that is presented in the standard. The FUNCTIONAL NIRS PHANTOM is composed of a material that has scattering characteristics similar to human tissues. Light intensity is mainly attenuated by scattering in the FUNCTIONAL NIRS PHANTOM. The amount of light transmission is varied with the (variable) optical aperture inside the FUNCTIONAL NIRS PHANTOM.

The specifications include the size, the reduced scattering and absorption coefficients, surface roughness, and material of the attenuators; the position and size of internal apertures; and the position of the optode to be attached. The phantom prototype implemented in this paper satisfies structural and material specifications. In addition, the FUNCTIONAL NIRS PHANTOM is required to have an OPTICAL LOSS of greater than 40 dB in state A in case of the performance test for PATHLENGTH-DEPENDENT HEMOGLOBIN CHANGE. The light attenuation change between states A and B is to be within 3 dB to 4 dB at each wavelength.

The OPTICAL LOSS of a FUNCTIONAL NIRS PHANTOM is defined in the Annex BB 3.3 of the standard as the ratio of the total power exiting a circular aperture on the exit side of specified diameter (8 mm) and the power injected on the entrance side $P_{\text{in}}$:

$$\mathrm{OL}=\frac{P_{\mathrm{ex},\text{total}}}{P_{\text{in}}},$$ (1)

where *OL* denotes the OPTICAL LOSS. $P_{\mathrm{ex},\text{total}}$ is the total power emitted from the entire exit aperture area into the full half space. Equation (1) is formula (BB. 9) of the standard.

The FUNCTIONAL NIRS PHANTOM specification is found in Annex BB 3.2 of the standard and is quoted in part below. Some descriptions have been slightly modified, such as deleting references to other parts of the standard. Please refer to the standard for the exact specifications.

• Ensure that each of the two parts of the attenuating material is greater than or equal to 60 mm in diameter, the thickness of each part is greater than or equal to 10 mm, and the total thickness of the two parts is greater than or equal to 30 mm.
• Ensure that the attenuating material has the following optical properties:
  • reduced scattering coefficient $\mu_{s}^{\prime }$: 0.8 to $1.2\;{\mathrm{mm}}^{-1}$
  • absorption coefficient $\mu_{a}$: 0.00 to $0.01\;{\mathrm{mm}}^{-1}$.
• POM or any other homogeneous, optically isotropic, and turbid material can be used, provided it meets these conditions.
  • Once the scattering and absorbing material is selected, the required OPTICAL LOSS can be adjusted by changing the thickness of the two attenuators or the size of the internal aperture, or both.
  • The OPTICAL LOSS can be increased by inserting a neutral density filter on the entrance face of the phantom. In particular, a phantom with an OPTICAL LOSS of 60 dB as required by some of the performance tests can be obtained using a 40 dB phantom and inserting an appropriate neutral density filter between the EMITTER OPTODE and the entrance face.
• The surface of the attenuating material should be finished with a roughness less than 1.6  μm.
• Ensure that the aperture on the entrance side that holds the EMITTER OPTODE is centered on the axis of the cylindrical material.
• Ensure that the materials of the variable optical aperture and the case enclosing the scattering materials of the FUNCTIONAL NIRS PHANTOM have little reflection at any relevant wavelength.
• Ensure that the diameter of the changeable internal aperture is less than 8 mm.
• Ensure that the changeable circular aperture is inserted between the two parts and is aligned with the centers of the apertures on the entrance and exit faces holding the EMITTER OPTODE and DETECTOR OPTODE.
• Ensure that the exit aperture has a diameter of 8  mm±0.1  mm and is centered on the axis of the cylindrical material.
• In state A, ensure that the FUNCTIONAL NIRS PHANTOM has an OPTICAL LOSS of greater than 40 dB. A changeable optical aperture sandwiched between two attenuating parts allows for the attenuation change between states A and B.
• Ensure that the light attenuation change between states A and B is between 3 and 4 dB at each wavelength of the me equipment.

### 6.3 Derivation of PATHLENGTH-DEPENDENT HEMOGLOBIN CHANGE

Functional NIRS derives changes in oxy- and deoxyhemoglobin concentrations associated with brain activation from changes in detected light intensities. The standard refers to these concentration changes as PATHLENGTH-DEPENDENT HEMOGLOBIN CHANGE. The process to derive the PATHLENGTH-DEPENDENT HEMOGLOBIN CHANGE is described in Annex BB. 2 of the standard. Below is a shortened description of the derivation. For a more detailed description, please refer to the standard. Equations (2)–(5) correspond to formulas (BB.2), (BB.3), (BB.4), and (BB.5) in Annex BB 3.1 of the standard, respectively.

According to the modified Beer-Lambert law, if the hemoglobin concentrations change from state A to state B, and $I_{A}(\lambda_{i})$ and $I_{B}(\lambda_{i})$ represent the detected light intensities corresponding to each state at wavelength $\lambda_{i}$, the ratio of $I_{B}(\lambda_{i})$ to $I_{A}(\lambda_{i})$ is related to the change in the attenuation $\Delta A(\lambda_{i})$ by the following equation.

$$\Delta A(\lambda_{i})=\log_{10}[\frac{I_{A}(\lambda_{i})}{I_{B}(\lambda_{i})}],$$ (2)

$$\Delta A(\lambda_{i})=\varepsilon_{O_{2}\mathrm{Hb}}(\lambda_{i})\Delta c_{O_{2}\mathrm{Hb}}L+\varepsilon_{O_{2}\mathrm{Hb}}(\lambda_{i})\Delta c_{O_{2}\mathrm{Hb}}L,$$ (3)

where Δ represents the difference between states B and A, the mean optical pathlength in the tissue L is assumed to be independent of wavelength and time during the measurement, $c_{O_{2}\mathrm{Hb}}L$ and $c_{\mathrm{HHb}}L$ are the oxyhemoglobin and deoxyhemoglobin concentrations, respectively, and $\varepsilon_{O_{2}\mathrm{Hb}}(\lambda_{i})$ and $\varepsilon_{\mathrm{HHb}}(\lambda_{i})$ are the corresponding molar decadic (i.e., base 10) absorption coefficients. By measuring $\Delta A(\lambda_{i})$ at two or more wavelengths, the PATHLENGTH-DEPENDENT HEMOGLOBIN CHANGE, $\Delta c_{O_{2}\mathrm{Hb}}L$ and $\Delta c_{\mathrm{HHb}}L$, can be obtained. For example, using two wavelengths, $\lambda_{1}$ and $\lambda_{2}$, $\Delta c_{O_{2}\mathrm{Hb}}L$ and $\Delta c_{\mathrm{HHb}}L$, are given by Eqs. (4) and (5), respectively:

$$\Delta c_{O_{2}\mathrm{Hb}}L=\frac{\varepsilon_{\mathrm{HHb}}(\lambda_{2})\Delta A(\lambda_{1})-\varepsilon_{\mathrm{HHb}}(\lambda_{1})\Delta A(\lambda_{2})}{\varepsilon_{O_{2}\mathrm{Hb}}(\lambda_{1})\varepsilon_{\mathrm{HHb}}(\lambda_{2})-\varepsilon_{O_{2}\mathrm{Hb}}(\lambda_{2})\varepsilon_{\mathrm{HHb}}(\lambda_{1})},$$ (4)

$$\Delta c_{\mathrm{HHb}}L=\frac{\varepsilon_{O_{2}\mathrm{Hb}}(\lambda_{1})\Delta A(\lambda_{2})-\varepsilon_{O_{2}\mathrm{Hb}}(\lambda_{2})\Delta A(\lambda_{1})}{\varepsilon_{O_{2}\mathrm{Hb}}(\lambda_{1})\varepsilon_{\mathrm{HHb}}(\lambda_{2})-\varepsilon_{O_{2}\mathrm{Hb}}(\lambda_{2})\varepsilon_{\mathrm{HHb}}(\lambda_{1})}.$$ (5)

## Data Availability

The data that support the findings of this study are available from the corresponding author upon reasonable request.
